# Association between height growth patterns in puberty and stature in late adolescence: A longitudinal analysis in chinese children and adolescents from 2006 to 2016

**DOI:** 10.3389/fendo.2022.882840

**Published:** 2022-07-22

**Authors:** Li Chen, Binbin Su, Yi Zhang, Tao Ma, Jieyu Liu, Zhaogeng Yang, Yanhui Li, Di Gao, Manman Chen, Ying Ma, Xijie Wang, Bo Wen, Jun Jiang, Yanhui Dong, Yi Song, Jun Ma

**Affiliations:** ^1^ Institute of Child and Adolescent Health, School of Public Health, Peking University; National Health Commission Key Laboratory of Reproductive Health, Beijing, China; ^2^ Institute of Population Research, Peking University Asia-Pacific Economic Cooperation (APEC) Health Science Academy, Beijing, China; ^3^ Vanke School of Public Health, Tsinghua University, Beijing, China; ^4^ School of Public Health and Preventive Medicine, Monash University, Melbourne, VIC, Australia; ^5^ Department of Atmospheric and Oceanic Science, Earth System Science Interdisciplinary Center, University of Maryland, College Park, MD, United States

**Keywords:** pubertal growth patterns, adult height, overweight and obesity, timing, intensity, duration

## Abstract

**Introduction:**

The relationship between the characteristics of puberty growth and the stature (height and overweight and obesity) in late adolescence was not clear. We aimed to explore the effects of puberty growth patterns on the stature in late adolescence.

**Methods:**

A total of 13,143 children from a longitudinal cohort from 2006 to 2016 in Zhongshan city of China were included. The Preece–Baines growth curve was fitted for each individual child, and the age at peak height velocity (APHV), peak height velocity (PHV), and age at take-off (TOA) were obtained from the Preece–Baines model. To compare the difference in height in late adolescence (at 18 years old) at different pubertal height growth patterns (height spurt timing, intensity, and duration), the height at baseline was matched by using the propensity score matching. The log-binomial model was applied to assess the association between the three pubertal height growth patterns (timing, intensity, and duration) and overweight and obesity status in late adolescence, controlling the urbanity and body mass index (BMI) at baseline.

**Results:**

After matching the baseline height, boys and girls in three pubertal patterns with early timing (*P* < 0.01), small intensity (*P* < 0.01), and short duration (*P* < 0.01) of height spurt had the lowest final height in the late adolescence. A 16% increase and 45% increase of risk for overweight and obesity were significantly associated with the early APHV in boys and girls, respectively, *relative risk* (*RR*) in boys, 1.16(95% confidence interval, CI: 1.03–1.30), *P* = 0.011; *RR* in girls, 1.45(1.21–1.75), *P* < 0.001. *A* 21% increase and 59% increase of risk for overweight and obesity were significantly associated with small PHV in boys and girls, respectively, *RR* in boys, 1.21(1.07–1.36), *P* < 0.001; *RR* in girls, 1.59(1.30–1.95), *P* < 0.001; and an 80% increase of risk for overweight and obesity with small spurt duration in girls (*RR* = 1.80; 95% CI: 1.49, 2.18; *P* < 0.001).

**Conclusion:**

Pubertal growth patterns, including earlier puberty onset timing, smaller puberty intensity, and shorter puberty spurt duration, had a positive association with lower height risks and higher overweight and obesity risks in late adolescence.

## Introduction

Puberty is an important period that comprised a series of physical and psychosocial changes during the transition from childhood to young adulthood (1). In this period, children and adolescents experience the rapid growth in stature and development of secondary sexual characteristics (2). The onset of puberty, also known as the beginning of puberty timing, was identified as an important public health indicator for children and adolescents due to its close association with multiple health outcomes (3). Previous study found that the early onset of puberty was associated with many adverse health consequences both in adolescence and adulthood, including obesity, type 2 diabetes, and other cardiovascular diseases (4).

The height growth patterns in puberty were always a key focus of adolescent health, and many methods of evaluating puberty growth that targeted physical height changes were proposed, such as pubertal development scale (PDS), sexual maturity ratings (SMRs) scales, and height growth patterns. The PDS is a self-reported measure of physical development for youth, which includes five dimensions: body hair, facial hair, voice change, skin change, and growth spurt and could result in misclassification of pubertal development due to self-reported data (5, 6). The SMR scales developed by Marshall and Tanner were used to evaluate the development of secondary sexual characteristics in children and adolescents (7–9). However, there were two potential limitations of SMRs including partial estimation and privacy, because the examiner could be highly subjective when determining the different stages of puberty timing and involved privacy when checking for secondary sexual characteristics in children and adolescents. The height growth patterns were widely used to evaluate puberty development due to its subjectivity and convenience (10, 11). The pubertal height growth patterns consisted of the phases of acceleration, followed by deceleration, and the eventual cessation of height growth, and its characteristics included the onset of growth acceleration (age at take-off), age at the peak height velocity (APHV), and duration of height growth.

The height growth patterns in puberty reflected the potentials of height and weight in children and adolescents, but whether it affected the end stature in early adulthood (that is late adolescence) has not been studied. Height and weight reflected long-term and, relatively, short-term nutritional status in a population. Previous studies showed a controversial association between puberty growth patterns and height in adulthood (10, 12). Cohort study showed that the characteristics of puberty growth patterns were not associated with the final height in early adulthood (10), but another longitudinal study found that the increase of height was widely attributed to puberty growth during the puberty (12). Similarly, with increasing attention to obesity, in addition to final height, the weight at early adulthood was also an important health outcome of adolescent height growth patterns. Overweight and obesity in childhood, adolescence, and late adolescence increased the risk of cardiovascular diseases in adulthood (13–15), and the overweight/obesity has also associated with psychosocial problems (e.g., poor quality of life and high psychological distress) (16–19). However, the association between puberty growth patterns and obesity was investigated in some studies, which also revealed controversial results that adults with overweight and obesity in childhood were associated with the earlier or delayed puberty timing (20). The above controversial results between the height growth patterns in puberty and later height and weight might be due to variation in different population, study designs, evaluation methods, or massive confounders. Few studies tried to use the longitudinal and intensive height follow-ups to explore the effects of height growth patterns in puberty on adult stature, including final height and overweight in early adulthood.

Timing, intensity, and duration of height spurt, which were represented by the APHV, peak height velocity (PHV), and the duration from beginning of the height spurt to the peak height growth velocity, were used to define the height growth patterns (21, 22). Previous studies indicated that deviation from the normal body mass index (BMI) status was associated with the timing of height spurt, and the association could be different in boys and girls (23–25). Thus, we used a longitudinal study to characterize pubertal height development patterns in three dimensions, namely, the timing, intensity, and duration in Chinese children and adolescents. Besides, we also tried to explore the effects of height growth patterns in puberty on the final height and overweight and obesity in late adolescence.

## Materials and methods

### Study design

The longitudinal data of the cohort came from the physical examination database in Zhongshan city of Guangdong province between 2006 and 2016 (26). The physical examination survey, which covered the school-aged students aged 7 to 8 years in Zhongshan city at baseline, was used to generate the longitudinal dynamic cohort with 11 years’ follow-up. The information about the longitudinal dynamic cohort has been mentioned in the previous study (27). The end point of follow-up was determined when they grew up at 17–18 years and finished the compulsory education stage. Thus, the survey in the city was close to the census of local children and adolescents except for the dropouts. Follow-up was conducted once every year with a relatively fixed survey date (from September 1 to October 31). The implementation of each follow-up survey was completed in strictly accordance with the requirements of the Chinese National Survey on Students’ Constitution and Health (CNSSCH) (28). Students (3,198) with missing data (height and weight) and severe dysfunction of vital organs, abnormal development of the spine, abnormal development of the limbs, and disabled were excluded, and the excluded sample was tested by conducting Little’s MCAR test (*P* > 0.05) and showed that the loss was by random. Finally, a total of 13,143 students who completed the 11-year follow-up were included in the dynamic cohort (shown in flow chart of **Supplementary Figure 1**). This study was approved by the Medical Research Ethics Committee of Peking University Health Science Center (reference number: IRB00001052-20033).

### Anthropometric measurements

All students in annual physical examination survey underwent complete medical examinations and measurements conducted by trained and qualified medical physicians from medical establishments. Height was measured by a portable stadiometer (model TZG, Jiangyin No. 2 Medical Equipment Factory, Jiangsu Province, China). The portable stadiometer was calibrated every day during the measurement using a steel ruler to measure the height value of the scale corresponding to the surface of the reference plate of the height gauge. The lever type weight scale (model RGT-140, Shanghai Dachuan Electronic Weighing Apparatus Co. Ltd, Shanghai, China) was used to measure the weight. The lever type weight scale was calibrated by using 10 and 20 kg standard weights placed on the scale. The standardized measurement procedure of height and weight referred to the anthropometry methods in the 2006 WHO Child Growth Standards (http://www.who.int/childgrowth/standards/en/). Height was measured to the nearest 0.1 cm, and weight was measured to the nearest 0.1 kg. Students were required to remove their shoes and stand on an altimeter with bare feet. The height and weight measurements were repeated twice, and the mean of two measurements was used as the final height and weight. The height and weight were recorded with one decimal (e.g., 123.9 cm and 35.9 kg).

BMI was calculated based on weight and height. The BMI definitions of the Working Group on Obesity in China (WGOC) were used to categorize the BMI groups: overweight and obesity (29). The age of late adolescence was defined as above 17 years old, whereas the age of pre-adolescence was defined as less than 10 years old. In the current study, the baseline age of all participants was less than 10 years, so we used the baseline BMI as the BMI in pre-adolescence, and all participants were older than 17 years in 2016, so we used the BMI in 2016 as the BMI in late adolescence. The BMI difference was defined as BMI in late adolescence minus BMI in pre-adolescence. Sociodemographic variables, including gender, date of birth, and urbanity (urban and rural), were collected at the first medical examination. The age of each survey was calculated by (date of examination-date of birth)/365.25.

### Fitting the preece–baines growth curve

The Preece and Baines model (PB) was fitted for each individual using nonlinear least squares and, mostly, longitudinal series of data (30–37). The model was widely used to determine the timing and tempo of growth events of anthropometric characteristics at puberty and until the measured values reached the final or adult size (30–32, 34, 35). The PB model was widely reported to fit the height growth in American and Asian countries, such as the United States (38), Mexico (39), India (34, 40), and China (41, 42). Separate models were fitted for each individual, and the analytical solution for velocity and acceleration was used to estimate the parameters of height spurt. In our study, the height at 17 and18 years old was considered to be the final height. The APHV, peak height velocity (PHV), and age at take-off (TOA) were obtained from the PB model. The APHV was defined as the age at the maximum height velocity. The PHV was defined as the velocity at the maximum spurt velocity. The TOA was defined as the age at the lowest height velocity when entering the height spurt. More information about fitting the PB growth curve was expressed in supplementary files (**Appendix** section).

### Evaluation of height growth patterns in puberty

The height spurt timing, intensity, and duration were used to comprehensively evaluate the pattern of height growth in puberty and were represented by APHV, PHV, and APHV minus TOA, respectively. Timing: The height spurt timing was represented by APHV, and the timing of height spurt was categorized into three groups: early (APHV<P_25_), medium (P_25_≤APHV≤ P_75_), and late (APHV>P_75_). Intensity: The PHV was used to represent the height spurt intensity. The intensity of height spurt was categorized into three groups: small (PHV<P_25_), medium (P_25_≤PHV≤P_75_), and large (PHV>P_75_). Duration: The height spurt duration was defined as the time from the beginning of the height spurt to the maximum height growth velocity (21). The height spurt duration was calculated by using APHV minus TOA, and the duration represented an indicator for growth spurt duration or pubertal duration (22): short (duration<P_25_), medium (P_25_≤duration≤P_75_), and long (duration>P_75_).

### Statistical analysis

Descriptive statistical methods were used to summarize the study population. Participants’ characteristics were summarized using mean (SD) for continuous variables and counted with percentages (%) for categorical variables. The t-test was used to evaluate the difference between continuous variables, and the χ^2^ test was performed for categorical variables.

To compare the difference in height at 18 years old in different height growth patterns in puberty (height spurt timing, intensity, and duration), excluding the influence of heredity and prophase nutrition, the height at baseline for all the participants was matched by using the propensity score matching (43). The generalized additive model was used to fit the height growth rate curve and height growth curve for different height growth patterns in puberty. Take three groups of height spurt timing (early, medium, and late) as an example. There were three potential effects of interest: early versus medium, medium versus late, and early versus late. For each contrast, a propensity score was estimated by logistic regression. Caliper-based 1:1 nearest neighbor matching was used to match the propensity score. The common-referent approach was used to perform matching. The medium group was considered as the referent group. We extracted students with the early or late group who had a common match of a student who was from the medium group. The link of the early or late group was conducted through their shared medium analog. We created a single cohort of these students and their medium group matches. A three-way-matched cohort was established using an algorithm. The algorithm required two generalized propensity scores for each student: probabilities of being suffered from the early and medium group, which together determined the probability of late group.

The association between the pattern of height spurt (timing, intensity, and duration) and BMI (or BMI change) in late adolescence used a multivariable linear regression model after adjustment for the urbanity and BMI in pre-adolescence. Two multivariable linear regression models were built with BMI in the late adolescence and BMI change as the dependent variables and the pattern of height spurt (timing, intensity, and duration) as the independent variables, separately. To estimate relative risk (RR), the log-binomial model was applied to assess the association between the pattern of height spurt (timing, intensity, and duration) and overweight and obesity (the overweight and obesity group was blended into one group) in late adolescence, after adjusting for the urbanity and BMI in pre-adolescence. There was abundant evidence to suggest that the association differed between boys and girls, therefore our analyses were conducted based on the stratified analysis by gender. R software (version 4.0.3) was used to perform all analyses in the study. Statistical significance was defined as a two-tailed *p*-value of less than 0.05.

## Results

### General characteristics of height growth patterns in puberty between boys and girls

**Figure 1:**
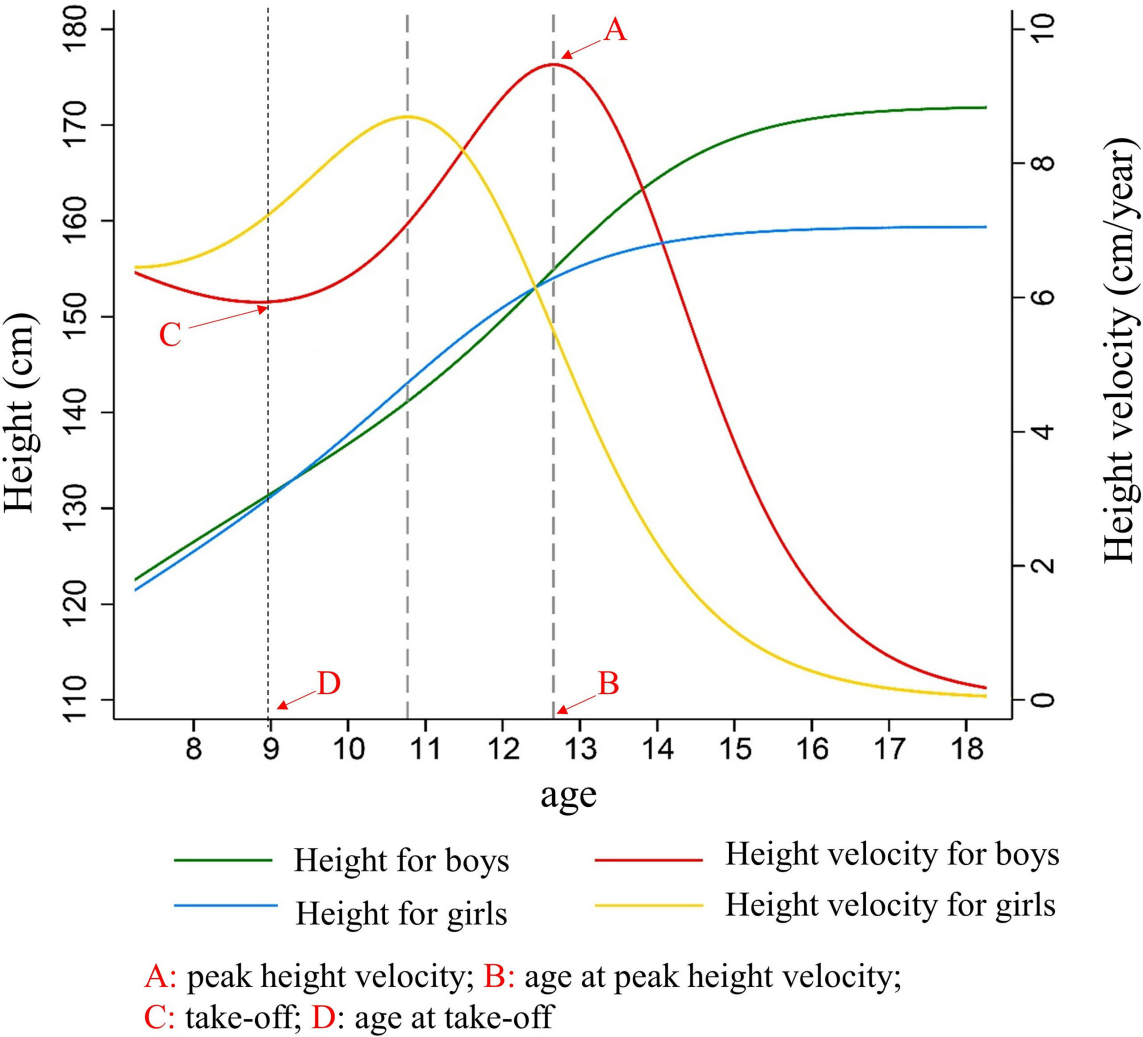
The characteristics of height growth patterns in puberty between boys and girls. Description of results in figure above: Because girls entered the height spurt earlier and the height growth rate was higher than that of boys at the same period. Girls began to catch up in height and surpassed boys at 9.2 years old, forming the first height crossover. As boys began to enter the height spurt, the growth rate of height accelerated, and the duration and PHV of height spurt exceeded that of girls, so that the height of boys exceeded that of girls at age of 12.6 years, forming the second crossover of height. After that, the height gap between boys and girls continued to expand, and the height of the boys in the late adolescence (171.8 ± 6.1 cm) was significantly higher than that of girls (159.45 ± 5.8 cm) (*t* = 117.39, *P* < 0.001).

A total of 13,143 students were included in the cohort study, including 7,729 boys (58.8%) and 5,414 girls (41.2%), 7,729 urban (68.8%) and 5,414 rural children (41.2%). The average follow-up duration was 9.0 ± 1.0 years. The height growth curve showed that the height of girls increased rapidly earlier than that of boys. The TOA of the girls was 7.9 ± 1.4 years old, which was 1.2 years earlier than that of the boys (*P* < 0.001). The APHV of the girls was 10.8 ± 1.0 years old, which was 1.9 years earlier than that of the boys (*P* < 0.001). The height surge of the girls was almost at 13.8 ± 2.4 years old, with 2.3 years earlier than that of the boys (16.1 ± 1.4 years old) (*P* < 0.001). The height spike duration of the boys was 3.6 ± 1.0 years with 0.7 years longer than that of the girls (2.9 ± 1.3 years, *P* < 0.001) (shown in **Table 1** and **Figure 1**).

**Table 1:** Characteristics for participants by gender.

	Boys (*N* = 7729)	Girls (*N* = 5414)	*t*	*P*
Residence, %				
Urban	4500(58.2)	3229(59.6)	4.74	0.030
Rural	3229(41.8)	2185(40.4)		
Follow-up years	9.1 ± 1.0	9.0 ± 1.0	4.84	<0.001
Age	8.1 ± 0.8	8.2 ± 0.9	4.17	<0.001
Height, cm	123.7 ± 5.2	122.6 ± 5.5	9.22	<0.001
Weight, kg	26.1 ± 5.5	25.0 ± 5.2	11.39	<0.001
BMI, kg/m^2^	16.1 ± 2.2	15.5 ± 1.9	16.83	<0.001
TOA, years	9.1 ± 1.4	7.9 ± 1.4	44.52	<0.001
APHV, years	12.7 ± 0.9	10.8 ± 1.0	106.90	<0.001
EA, years	16.1 ± 1.4	13.8 ± 2.4	57.34	<0.001
Duration of the growth spurt, years	3.6 ± 1.0	2.9 ± 1.3	34.58	<0.001
Pre-pubertal height, cm	123.7 ± 5.2	122.6 ± 5.5	9.22	<0.001
Height at peak height velocity, cm	154.9 ± 6.5	142.4 ± 6.8	105.78	<0.001
Post pubertal height, cm	171.8 ± 6.1	159.4 ± 5.8	117.39	<0.001
PHV, cm/year	9.6 ± 1.4	8.7 ± 2.0	25.87	<0.001

*TOA, age at take-off; APHV, age at peak height velocity; EA, age at the growth spurt ends;PHV, peak height velocity.*

### The timing of height spurt and final height in late adolescence

**Figure 2:**
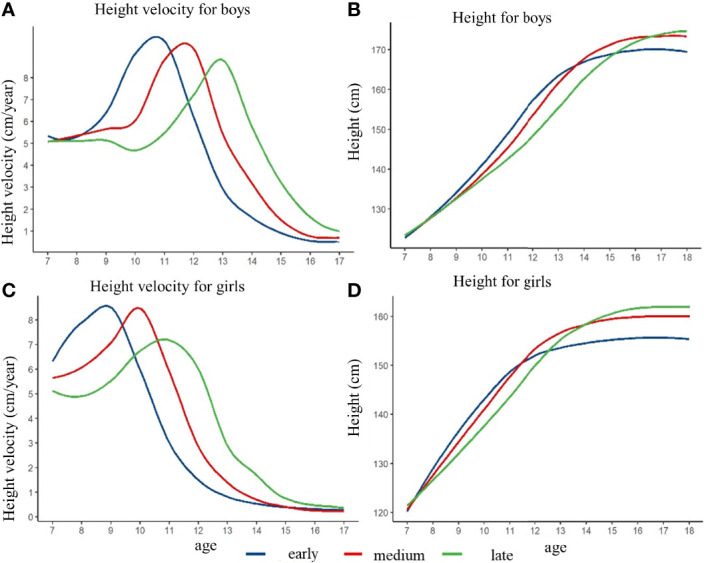
The height growth trajectory of boys and girls with different height spurt timing (APHV) after matching the pre-pubertal height at baseline. **(A)** height velocity for boys; **(B)** height for boys; **(C)** height velocity for girls; **(D)** height for girls.

**Figure 2** illustrated the height growth rate curve and height growth curve of children with different height spurt timing in pre-pubertal normal-weight children fitted by the generalized additive model. Three groups (early group, normal group, and late group) were determined based on the height growth rate curve. In boys, the APHV in the height spurt early group was 10.7 years old, which was earlier than that in the normal group (11.7 years old) and the late group (12.9 years old). The PHV of the early group was 9.9 cm/year, which was higher than that of the normal group (9.6 cm/year) and the late group (8.8 cm/year). In girls, the APHV of the height surge group was 8.8 years, which was earlier than that of the normal group (9.9 years) and the late group (10.8 years). PHV of the early group was 8.6 cm/year, which was higher than that of the normal group (8.5 cm/year) and the late group (7.2 cm/year) (shown in **Figure 2** and **Supplementary Table 1**). After controlling for pre-pubertal height at baseline, the height growth curve of children with different height spurt timing was different. The group with late height spurt timing had the highest height in late adolescence, and the group with early timing had the lowest height in late adolescence. The height difference in late adolescence was more pronounced among girls. Different results might exist if baseline height was not matched, which could derive the opposite conclusion (shown in **Supplementary Figure 2** and **Supplementary Table 2**).

### The intensity of height spurt and final height in late adolescence

**Figure 3:**
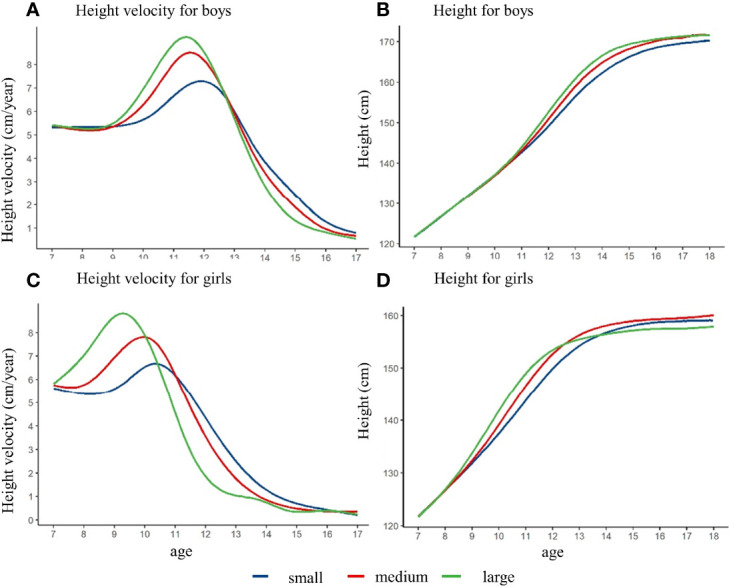
The height growth trajectory of boys and girls with different height spurt intensity (PHV) after matching the pre-pubertal height at baseline. **(A)** height velocity for boys; **(B)** height for boys; **(C)** height velocity for girls; **(D)** height for girls.

**Figure 3** showed that the height growth curve and height growth rate curve of children with different height spurt intensity, after controlling the pre-pubertal height at baseline. The height of the girls in the large intensity group was slightly lower than that in the small and medium group at the age of 18 years. The height of the boys in different intensity groups did not have significant difference. Among the boys, the PHV of the small, medium, and large intensity group was 7.3 cm/year, 8.5 cm/year, and 9.2 cm/year, respectively. The APHV of the small group was 11.9 years old, which was higher than that of the medium group (11.6 years old) and the early group (11.4 years old). For the girls, the PHV of small, medium, and large intensity group was 6.7 cm/year, 7.8 cm/year, and 8.8 cm/year, and the APHV was 10.3 years old, 9.9 years old, and 9.3 years old for small, medium, and large intensity group (shown in **Supplementary Figure 3** and **Supplementary Table 3**). Different results might exist if baseline height was not matched, which could derive the opposite conclusion (shown in **Supplementary Figure 3** and **Supplementary Table 4**).

### The duration of height spurt and final height in late adolescence

**Figure 4:**
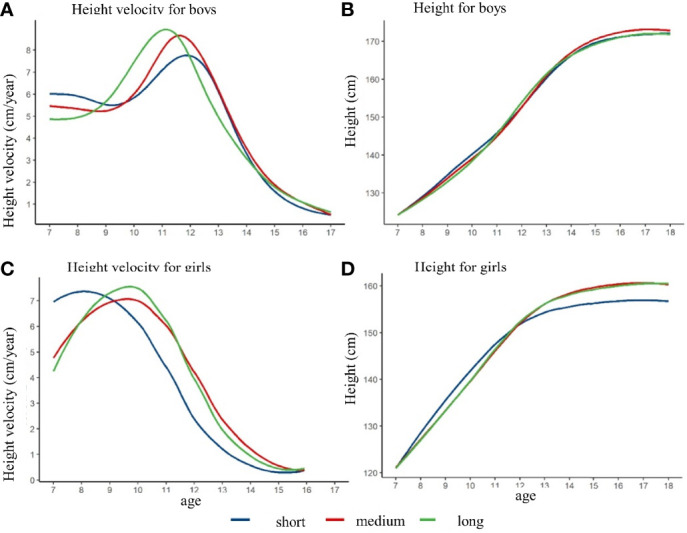
The height growth trajectory of boys and girls with different height spurt duration after matching the pre-pubertal height at baseline. **(A)** height velocity for boys; **(B)** height for boys; **(C)** height velocity for girls; **(D)** height for girls.

After controlling the pre-pubertal height at baseline, the height growth curve and height growth rate curve with different height spurt duration were shown in **Figure 4**. The height of the girls in the short-duration group was lower than that in the medium and long duration group at age of 18 years. The height of the boys in these three duration groups were not observed a significant difference. The PHV of the short, medium, and long duration group was 7.8 cm/year, 8.7 cm/year, and 8.9 cm/year among the boys, respectively. The APHV was 11.8 years old, 11.5 years old, and 11.2 years old for short, medium, and long duration group among the boys. Among the girls, the PHV of the short, medium, and long duration group was 7.4 cm/year, 7.1 cm/year, and 7.5 cm/year, respectively. The APHV of the short group was 8.1 years old, which was lower than that of the medium group (9.7 years old) and the long group (9.7 years old) (shown in **Supplementary Table 5**). Similarly, different results might exist if baseline height was not matched, which could derive the opposite conclusion (shown in **Supplementary Figure 4** and **Supplementary Table 6**).

### Association between height growth patterns and overweight and obesity in late adolescence

**Table 2** showed the association between characteristics of height growth patterns and BMI in late adolescence. After controlling for urbanity and BMI before puberty, the BMI in late adolescence for boys decreased by 0.34 kg/m^2^ with the 1-year delay of APHV (β = −0.34; 95% CI: −0.40, −0.27; *P* < 0.001), 0.04 kg/m^2^ with the 1 cm/year increase of PHV (β = −0.04; 95% CI: −0.06, −0.03; *P* < 0.001), and 0.13 kg/m^2^ with 1 year increase of height spurt duration (β = −0.13; 95% CI: −0.19, −0.07; *P* < 0.001). For the girls, the BMI in late adolescence decreased by 0.12 kg/m^3^ with the 1-year delay of APHV (β = −0.12; 95% CI: −0.18, −0.06; *P* < 0.001), 0.01 kg/m^2^ with the 1 cm/year increase of PHV (β = −0.01; 95% CI: −0.02, 0.01; *P* = 0.375), and increased by 0.01 kg/m^2^ with 1-year increase of height spurt duration (β = 0.01; 95% CI: −0.04, 0.05; *P* = 0.858). Similar associations were found between the change of BMI and APHV in both boys and girls. The BMI change for the boys decreased by 0.05 kg/m^2^ with the 1-year delay of APHV (β = −0.05; 95% CI: −0.08, −0.02; *P* < 0.001), 0.02 kg/m^2^ with the 1 cm/year increase of PHV (β = −0.02; 95% CI: −0.04, 0.01; *P* = 0.162), and 0.02 kg/m^2^ with 1-year increase of height spurt duration (β = −0.13; 95% CI: −0.05, 0.01; *P* = 0.084) among boys. For girls, the BMI change decreased by 0.14 kg/m^3^ with the 1-year delay of APHV (β = −0.14; 95% CI: −0.17, −0.11; *P* < 0.001), 0.13 kg/m^2^ with the 1 cm/year increase of PHV (β = −0.13; 95% CI: −0.15, −0.10; *P* < 0.001), and 0.13 kg/m^2^ with 1-year increase of height spurt duration (β = −0.13; 95% CI: −0.16, −0.10; *P* < 0.001).

**Table 2:** The association between characteristics of height growth patterns and body mass index (BMI) in late adolescence.

		Boys		Girls	
		β (95% CI)	*P*	β (95% CI)	*P*
BMI	APHV	−0.34(−0.40, −0.27)	<0.001	−0.12(−0.18, −0.06)	<0.001
	PHV	−0.04(−0.06, −0.03)	<0.001	−0.01(−0.02, 0.01)	0.375
	Height spurt duration	−0.13(−0.19, −0.07)	<0.001	0.01(−0.04, 0.05)	0.858
BMI change	APHV	−0.05(−0.08, −0.02)	<0.001	−0.14(−0.17, −0.11)	<0.001
	PHV	−0.02(−0.04, 0.01)	0.162	−0.13(−0.15, −0.10)	<0.001
	Height spurt duration	−0.02(−0.05, 0.01)	0.084	−0.13(−0.16, −0.10)	<0.001

**Figure 5:**
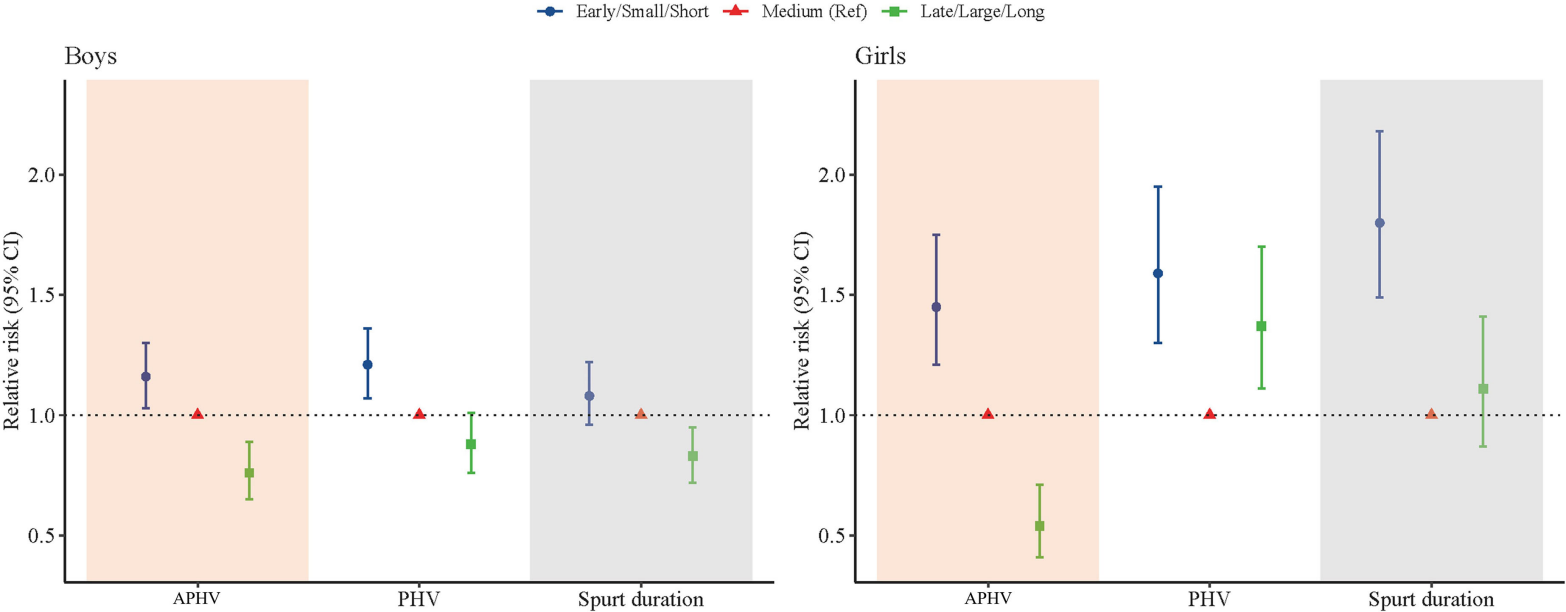
Association between height growth patterns and overweight and obesity in late adolescence of Chinese children and adolescents.

The association between the height growth patterns and overweight and obesity in late adolescence was shown in **Figure 5**. For the boys, we observed that a 16% increase of risk for overweight and obesity was significantly associated with the early APHV (*RR* = 1.16; 95% CI: 1.03, 1.30; *P* = 0.011), and a 21% increase of risk for overweight and obesity was significantly associated with small PHV (*RR* = 1.21; 95% CI: 1.07, 1.36; *P* < 0.001). For the girls, we observed that a 45% increase of risk for overweight and obesity was significantly associated with the early APHV (*RR* = 1.45; 95% CI: 1.21, 1.75; *P* < 0.001), a 59% increase of risk for overweight and obesity was significantly associated with small PHV (*RR* = 1.59; 95% CI: 1.30, 1.95; *P* < 0.001), and an 80% increase of risk for overweight and obesity was significantly associated with small spurt duration (*RR* = 1.80; 95% CI: 1.49, 2.18; *P* < 0.001).

## Discussion

To the best of our knowledge, this was the first and largest cohort study to reveal the height growth patterns in puberty for children and adolescents and to quantify its impact on final height and overweight and obesity in late adolescence in China. By nearly 11 years of follow-up, we found that the early APHV was significantly associated with a lower final height in both boys and girls after matching the characteristics at baseline. For the boys, we observed that the early APHV and small PHV were predictors for overweight and obesity in late adolescence, which increased the risk by 16% and 21%, respectively. For the girls, we found that early APHV, small PHV, and short spurt duration were predictors for overweight and obesity in late adolescence, which increased the risk by 45%, 59%, and 80%, respectively. This study could help to predict the future height potentials and overweight and obesity risk at adulthood based on their growth patterns during the puberty, which provided evidence for the early prevention and targeted interventions for short stature and overweight and obesity from the perspective of pubertal development in children and adolescents.

The present study found that the adolescents with a later APHV tended to have a higher height in 18 years, which was consistent with many previous studies (10, 44, 45). The Gothenburg Osteoporosis and Obesity Determinants (GOOD) cohort study in Sweden showed a positive correlation between APHV and adult height (*r* = 0.09, *P* = 0.03), especially with the length of lower limbs (*r* = 0.15, *P* < 0.001) (44). Another cohort study in the United Kingdom also found a significant correlation between APHV and bone growth, which showed that a later APHV was associated with greater growth of bone in adolescence (45). Besides, we found that PHV was associated with the adult height in boys and height spurt duration was associated with the adult height in girls. Consistent with our findings, Tao and colleagues found that a longer puberty period was significantly associated with a greater height growth using a cohort in Japan (46). Generally, adolescent height growth accounted for about 17%–18% of adult height, which corresponded to about 30–31 cm for boys and 28–29 com for girls, respectively (10). Together with previous studies, our studies showed that adolescent height spurt was a critical process of individual growth and was also one of the important factors that determined the adult height. Moreover, we found that there were substantial differences in the association between the height spurt and the adult height, which implied that the puberty height growth was controlled by a complex network comprising numerous genetic factors (47).

We found that APHV, PHV, and spurt duration were negatively associated with BMI in late adolescence, which was also consistent with previous studies (48–51). Sandhu and his colleagues used a cohort study and found that a decrease of 0.55 kg/m^3^ in late adolescence was associated with per 1 sd delay of APHV (51). The current study identified that APHV, PHV, and spurt duration were associated with overweight and obesity at early adulthood. Although some previous studies examined such association above using a different index, such as BMI z-score, it also similarly implied that the early onset of height spurts in children and adolescents might lead to an increased risk of overweight and obesity in late adolescence. However, the impact of PHV and spurt duration on the risk of overweight and obesity in early adulthood was still inconsistent among previous studies (50), which might be related to the family feeding environment (52). Besides, pre-adolescent nutritional status was a very important confounding factor, allowed for the relationship between height spurt and overweight and obesity in late adolescence (53). On the one hand, the nutritional status of individuals at different stages of life had significant continuity. For example, children who were obese were much more likely to remain obese in adulthood (26). On the other hand, the process of height spurt was also influenced by nutritional status during childhood, especially, pre-adolescence. Thus, it might mis-estimate the effects of the height growth patterns on the overweight and obesity in late adolescence if the pre-adolescent nutritional status was not adjusted in the analyses.

The underlying mechanisms behind the observed association between height spurt and stature in late adolescence were not understood well, and two potential pathways could be proposed. First, individuals with earlier APHV tended to have shorter adult height, and it might be caused by the differences in the timing of spurt of different parts of the body. It was found that APHV was associated with the length of limbs in adulthood (9, 51, 54). Second, the early onset of height puberty might lead to an increase in body fat content. Children with earlier APHV were observed to have a higher trajectory of body fat between 9 and 18 years (55). Generally, the early onset of height spurt was associated with reduced adult height and increased body fat, which might work together to increase the risk of overweight and obesity in late adolescence. The metabolism, accumulation, and distribution of adipose tissue, which may be impacted by an increase in sex hormone release during puberty and height spurt, might be the biological mechanisms behind the association between BMI and height spurt (5, 56–58). The increasing in testosterone that occurs throughout puberty has a substantial influence on adipose tissue distribution (59), even though the mechanism was still unclear and further studies were needed to verify such association.

Thus, the present study has several implications. First, we found, for the first time, that the three dimensions of puberty height spurt were associated with adult height, which indicated that the puberty period was the key period for the intervention of height development. Height growth patterns were independent of other factors, which could help to identify the vulnerable groups and adopt targeted interventions to prevent short stature in children and adolescents. Second, our study showed that puberty was the key window of intervention for overweight and obesity as well as cardiovascular diseases in adulthood. As adolescents aged 10 to 19 years accounted for more than 16% of the global population, interventions on overweight and obesity using scientific guidance of height growth patterns in children and adolescents would not only benefit themselves but also help to increase the well-being of the total population (60). Third, it was difficult to monitor puberty process as the sexual tests were private. However, our study indicated that families and schools could take continuous height measurements for children and adolescents, which could be easily implemented to monitor the individual’s puberty development. Fourth, puberty was not directly measured in this research. However, height spurt was utilized as a puberty signal. Future research should include more puberty indicators, such as sexual development and PDS. Fifth, the final height in this study may not be representative of adult height. Adolescents may continue to grow after the final height measurement. Our study has several strengths. First, with large sample size and a long follow-up period, this study enabled us to capture the detailed changes in the height spurt process to evaluate the height growth patterns in Chinese children and adolescents. Second, the cohort design used in this study could help us to establish a causal relationship between height growth patterns and stature in late adolescence with robust evidence for interventions. Third, we comprehensively evaluated the height spurt in three different dimensions, including timing, intensity, and duration, which could give a more precise and comprehensive estimation of the height growth patterns. However, there were also several limitations in the present study. First, the population was recruited in one single city, which might be not enough to represent the national population in China. However, there are certain parallels in children’s growth patterns across countries, regions, and ethnic groups, and the current study may provide some evidence for researchers in other locations and countries to investigate the growth pattern. Second, the interval between each follow-up was only 1 year, which could not provide a more precise estimation. However, our study included almost all the school-aged children and adolescents in Zhongshan city. With such a great sample size, it was unrealistic to carry out much more intensive follow-up. Finally, the health ID number, which was used to match data, cannot identify students who have transferred or dropped out.

In conclusion, our study examined the characteristics of puberty height spurt for children and adolescents and provided strong evidence that pubertal growth patterns, including earlier timing, smaller intensity, and shorter spurt duration, were predictive of lower risks of height and higher risks of overweight and obesity in late adolescence. The findings suggested that continuous height measurements should be taken for children and adolescents to identify the vulnerable group and adopt targeted interventions by appropriately delaying the onset of puberty, increasing the height spurt intensity and duration to increase the final height levels and prevent overweight and obesity risks in adulthood. More studies were still needed to verify the underlying mechanism.

## Data availability statement

The raw data supporting the conclusions of this article will be made available by the authors, without undue reservation.

## Ethics statement

The studies involving human participants were reviewed and approved by the Medical Research Ethics Committee of Peking University Health Science Center. Written informed consent to participate in this study was provided by the participants’ legal guardian/next of kin.

## Author contributions

LC and BS conceptualized and designed the study, completed the statistical analyses, drafted the initial manuscript, and reviewed and revised the manuscript; YD, JM, and YS contributed to the conceptualization and design of the study; supervised the data collection, the statistical analyses, and initial drafting of the manuscript; and reviewed and revised the manuscript; BW and JJ assisted with the statistical analyses and critically reviewed and revised the manuscript; TM, YZ, JL, ZGY, YL, DG, MC, YM, and XW assisted with the data processing, statistical analyses, and the interpretation of the data. All authors approved the final manuscript as submitted and agree to be accountable for all aspects of the work.

## Funding

The present study was supported by the National Natural Science Foundation (grant 2103865 to Yanhui Dong) and the project funded by the China Postdoctoral Science Foundation (BX20200019 and 2020M680266 to Yanhui Dong).

## Conflict of interest

The authors declare that the research was conducted in the absence of any commercial or financial relationships that could be construed as a potential conflict of interest.

## Publisher’s note

All claims expressed in this article are solely those of the authors and do not necessarily represent those of their affiliated organizations, or those of the publisher, the editors and the reviewers. Any product that may be evaluated in this article, or claim that may be made by its manufacturer, is not guaranteed or endorsed by the publisher
