# Supplementary material for: Association between height growth patterns in puberty and stature in late adolescence: A longitudinal analysis in chinese children and adolescents from 2006 to 2016
Source: Front Endocrinol (Lausanne). 2022 Jul 22;13:882840. doi: 10.3389/fendo.2022.882840 (PMC9354934; doi:10.3389/fendo.2022.882840)
Supplement: Supplementary file 1 [file DataSheet_1.docx]

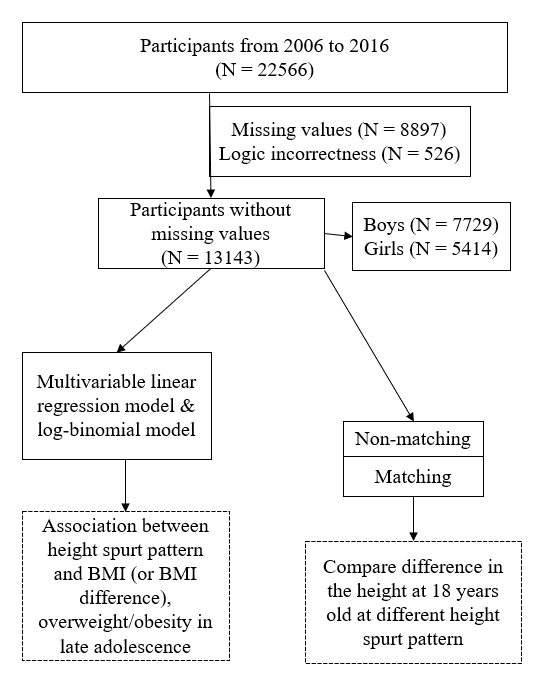


**Figure S1**. Data cleaning and analysis flow chart


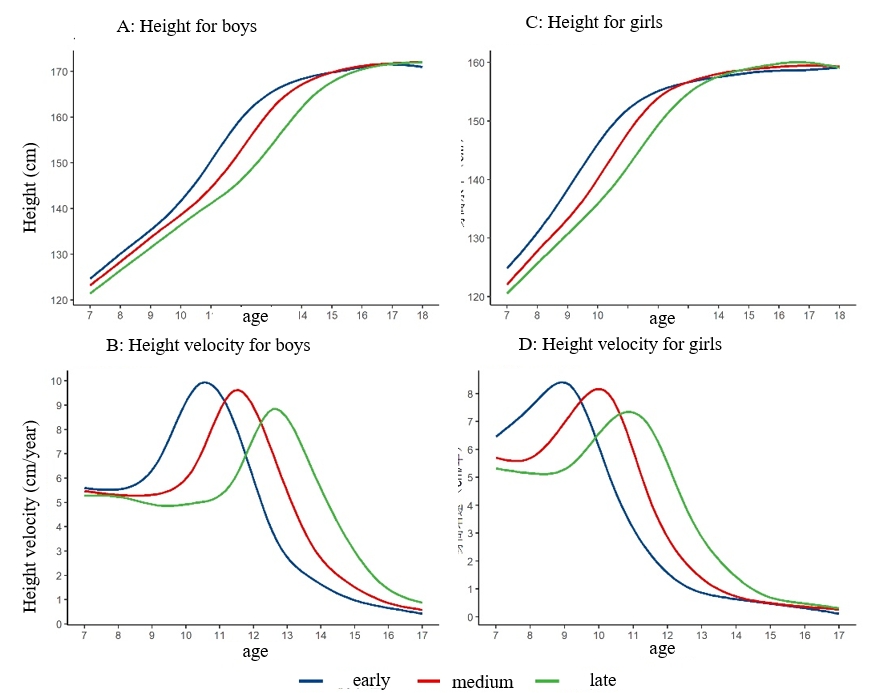


**Figure S2. The trajectory of height growth for boys and girls with different height spurt timing (before matching)**

**Table S1.** The characteristics of height spurt timing by boys and girls

| variables | Boys | | |  | | girls | | |  |
| --- | --- | --- | --- | --- | --- | --- | --- | --- | --- |
|  | early | medium | late | |  | early | medium | late | |
| Velocity at the begin of the spurt, cm/year | 5.3 | 5.1 | 5.1 | |  | 6.3 | 5.7 | 5.1 | |
| Velocity at the end of the spurt, cm/year | 0.5 | 0.7 | 1.0 | |  | 0.3 | 0.2 | 0.6 | |
| PHV, cm/year | 9.9 | 9.6 | 8.8 | |  | 8.6 | 8.5 | 7.2 | |
| APHV, years old | 10.7 | 11.7 | 12.9 | |  | 8.8 | 9.9 | 10.8 | |

Note: APHV, age at peak height velocity; PHV, peak height velocity.

**Table S2.** Comparison of the height of boys and girls aged from 7 to 18 years old with different height spurt timing

| Age | Boys | | | | girls | | | |
| --- | --- | --- | --- | --- | --- | --- | --- | --- |
|  | early | medium | late | *P* | early | medium | late | *P* |
| 7 | 123.1±5.7 | 123.1±5.8 | 123.1±5.8 | 1.000 | 121.1±2.6 | 121.1±2.6 | 121.1±2.6 | 0.999 |
| 8 | 128.4±4.6 | 127.9±4.8 | 127.8±4.8 | 0.743 | 128.2±3.2 | 127.5±3.1 | 127.0±3.0 | 0.122 |
| 9 | 133.3±5.4 | 133.3±5.1 | 133.1±4.8 | 0.952 | 135.2±3.9 | 133.2±3.5^*^ | 131.9±3.0^**^ | <0.001 |
| 10 | 139.8±5.3 | 138.6±4.8 | 138.0±5.2 | 0.143 | 143.4±3.7 | 140.0±4.0^**^ | 137.1±2.9^**^ | <0.001 |
| 11 | 148.8±6.5 | 144.6±5.3^**^ | 142.6±5.2^**^ | <0.001 | 149.3±3.0 | 148.2±4.0 | 143.2±3.6^**^ | <0.001 |
| 12 | 157.9±6.1 | 153.3±5.7^**^ | 147.9±5.7^**^ | <0.001 | 152.0±3.1 | 154.0±3.8^*^ | 150.2±4.3^*^ | <0.001 |
| 13 | 163.9±6.0 | 162.5±5.3 | 154.8±7.1^**^ | <0.001 | 153.7±3.5 | 156.8±3.9^**^ | 155.9±4.0^**^ | <0.001 |
| 14 | 166.8±5.7 | 167.8±5.2 | 163.5±7.0^**^ | <0.001 | 154.5±3.6 | 158.2±4.0^**^ | 158.7±4.3^**^ | <0.001 |
| 15 | 168.4±6.0 | 171.1±5.4^*^ | 168.8±6.1 | 0.019 | 155.0±3.8 | 158.9±4.5^**^ | 160.3±4.5^**^ | <0.001 |
| 16 | 169.1±6.0 | 172.4±5.5^**^ | 172.4±6.0^**^ | 0.001 | 155.4±3.9 | 159.3±4.4^**^ | 160.6±4.4^**^ | <0.001 |
| 17 | 169.9±5.6 | 172.8±5.3 | 173.0±5.3^*^ | 0.025 | 155.8±3.1 | 160.3±4.1^**^ | 162.4±4.0^**^ | <0.001 |
| 18 | 169.9±4.9 | 172.9±4.0^*^ | 173.5±3.0^**^ | 0.018 | 155.9±3.6 | 160.4±3.2^**^ | 162.5±5.2^**^ | <0.001 |
| Height difference | 46.8±8.2 | 49.8±6.8^*^ | 50.4±7.8^**^ | <0.001 | 34.8±6.0 | 39.3±4.8^**^ | 41.4±5.8^**^ | <0.001 |

Note:  ^*^ *P*<0.05，^**^ *P*<0.01, Height difference: height at 18 – the height at 7


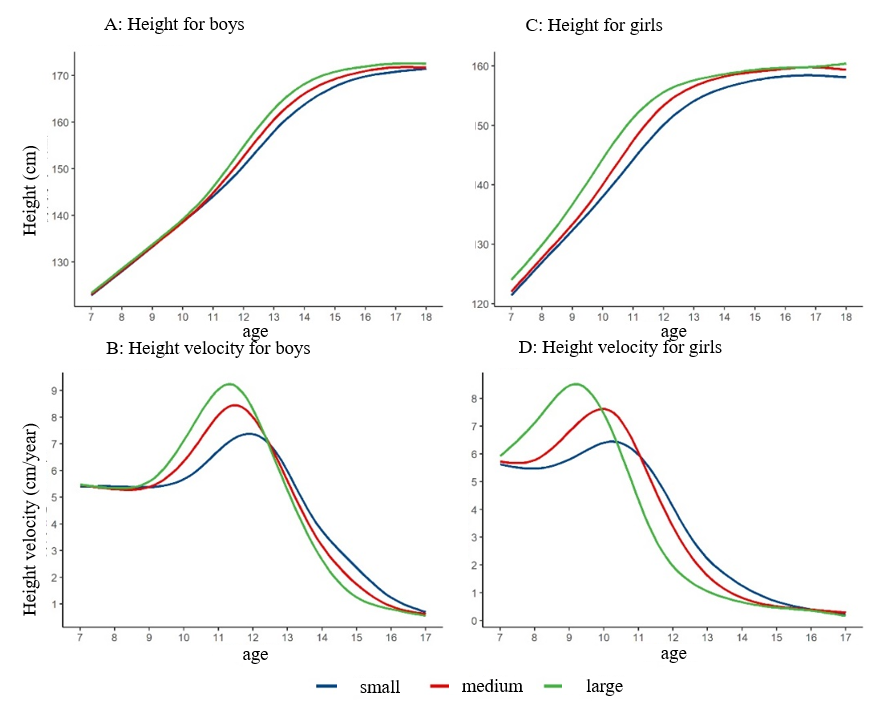


**Figure S3.** The trajectory of height growth for boys and girls with different height spurt intensity (before matching)

**Table S3.** The characteristics of height spurt intensity by boys and girls

| variables | Boys | | |  | girls | | |
| --- | --- | --- | --- | --- | --- | --- | --- |
|  | small | medium | large |  | small | medium | large |
| Velocity at the begin of the spurt, cm/year | 5.3 | 5.4 | 5.4 |  | 5.6 | 5.8 | 5.8 |
| Velocity at the end of the spurt, cm/year | 0.8 | 0.7 | 0.5 |  | 0.2 | 0.4 | 0.2 |
| PHV, cm/year | 7.3 | 8.5 | 9.2 |  | 6.7 | 7.8 | 8.8 |
| APHV, years old | 11.9 | 11.6 | 11.4 |  | 10.3 | 9.9 | 9.3 |

Note: APHV, age at peak height velocity; PHV, peak height velocity.

**Table S4.** Comparison of the height of boys and girls aged from 7 to 18 years old with different height spurt intensity

| Age | Boys | | | |  | Girls | | | |
| --- | --- | --- | --- | --- | --- | --- | --- | --- | --- |
|  | small | medium | large | *P* |  | small | medium | large | *P* |
| 7 | 121.6±4.2 | 121.7±4.1 | 121.7±4.0 | 0.957 |  | 121.8±3.7 | 121.8±3.7 | 121.8±3.7 | 1.000 |
| 8 | 126.7±4.6 | 126.8±4.4 | 126.8±4.5 | 0.887 |  | 126.7±4.2 | 126.8±4.1 | 126.7±4.1 | 0.978 |
| 9 | 131.9±4.8 | 131.8±4.8 | 131.9±4.7 | 0.862 |  | 132.0±4.4 | 132.2±4.4 | 133.7±5.3^**^ | 0.006 |
| 10 | 137.2±5.1 | 137.0±5.2 | 137.2±5.2 | 0.680 |  | 137.3±4.6 | 139.1±4.8^*^ | 141.8±6.3^**^ | <0.001 |
| 11 | 142.7±5.7 | 143.1±5.7 | 144.0±6.6^**^ | 0.001 |  | 143.8±4.9 | 146.6±5.2^**^ | 149.1±5.3^**^ | <0.001 |
| 12 | 149.1±6.4 | 150.9±6.7^**^ | 152.7±7.8^**^ | <0.001 |  | 150.0±4.7 | 152.8±4.6^**^ | 153.5±4.2^**^ | <0.001 |
| 13 | 156.5±6.7 | 159.0±6.5^**^ | 160.9±7.0^**^ | <0.001 |  | 154.3±4.4 | 156.3±4.2^**^ | 155.5±4.0 | <0.001 |
| 14 | 162.4±5.9 | 164.9±5.6^**^ | 166.6±5.6^**^ | <0.001 |  | 156.8±4.1 | 158.1±4.2^*^ | 156.4±4.2 | 0.002 |
| 15 | 166.1±5.3 | 168.2±5.3^**^ | 169.3±5.3^**^ | <0.001 |  | 158.0±4.4 | 158.9±4.1 | 157.2±4.3 | 0.005 |
| 16 | 168.6±5.4 | 170.1±5.3^**^ | 170.5±5.3^**^ | <0.001 |  | 158.8±4.4 | 159.4±4.4 | 157.3±4.3^*^ | 0.001 |
| 17 | 169.5±5.5 | 171.0±5.2^**^ | 171.3±5.4^**^ | <0.001 |  | 158.8±3.8 | 159.5±4.4 | 157.4±4.3 | 0.005 |
| 18 | 169.6±6.4 | 171.2±5.5^**^ | 171.5±6.8^**^ | <0.001 |  | 158.9±4.4 | 159.6±4.1 | 157.5±5.1 | 0.004 |
| Height difference | 48.0±7.2 | 49.8±6.1 | 49.8±7.7 | 0.613 |  | 37.1±6.2 | 37.8±5.1 | 35.7±7.0 | 0.523 |

Note:  ^*^ *P*<0.05，^**^ *P*<0.01, Height difference: height at 18 – the height at 7


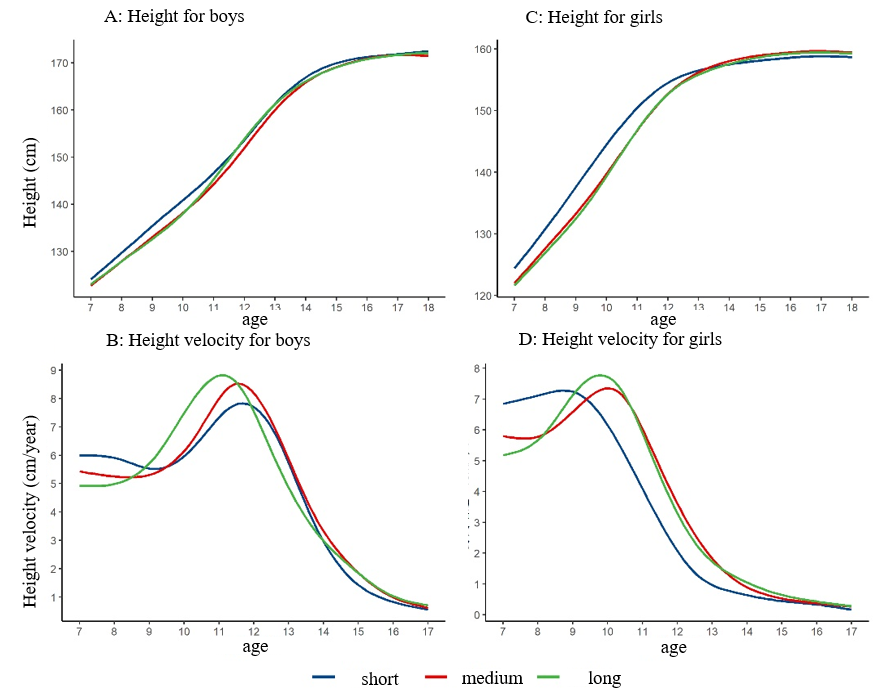


**Figure S4.** The trajectory of height growth for boys and girls with different height spurt duration (before matching)

**Table S5.** The characteristics of height spurt duration by boys and girls

| variables | Boys | | |  | Girls | | |
| --- | --- | --- | --- | --- | --- | --- | --- |
|  | short | medium | long |  | short | medium | long |
| Velocity at the begin of the spurt, cm/year | 6.0 | 5.5 | 4.9 |  | 7.0 | 4.8 | 4.3 |
| Velocity at the end of the spurt, cm/year | 0.5 | 0.5 | 0.6 |  | 0.3 | 0.4 | 0.4 |
| PHV, cm/year | 7.8 | 8.7 | 8.9 |  | 7.4 | 7.1 | 7.5 |
| APHV, years old | 11.8 | 11.5 | 11.2 |  | 8.1 | 9.7 | 9.7 |

Note: APHV, age at peak height velocity; PHV, peak height velocity.

Table S6. Comparison of the height of boys and girls aged from 7 to 18 years old with different height spurt duration

| age | Boys | | | |  | Girls | | | |
| --- | --- | --- | --- | --- | --- | --- | --- | --- | --- |
|  | short | medium | long | *P* |  | short | medium | long | *P* |
| 7 | 124.3±4.0 | 124.2±4.1 | 124.1±4.1 | 0.937 |  | 121.9±4.2 | 121.9±4.3 | 121.9±4.3 | 1.000 |
| 8 | 128.9±4.4 | 128.6±4.5 | 128.3±4.4 | 0.103 |  | 127.7±4.0 | 126.6±4.0 | 126.6±4.0 | 0.209 |
| 9 | 134.9±4.6 | 133.9±4.6^**^ | 132.9±4.5^**^ | <0.001 |  | 135.3±5.3 | 132.7±4.3^**^ | 132.1±4.4^**^ | <0.001 |
| 10 | 140.2±4.8 | 139.1±5.0^**^ | 138.4±5.0^**^ | <0.001 |  | 141.2±5.9 | 138.9±4.8^*^ | 138.8±5.0^*^ | 0.014 |
| 11 | 145.8±5.9 | 144.8±5.8^*^ | 145.4±6.5 | 0.051 |  | 147.8±5.6 | 146.1±5.6 | 146.6±5.7 | 0.181 |
| 12 | 152.6±7.1 | 152.6±7.1 | 154.0±7.0^*^ | 0.004 |  | 152.0±4.5 | 152.4±5.4 | 153.0±4.9 | 0.486 |
| 13 | 160.4±7.3 | 161.0±6.9 | 161.4±6.3 | 0.071 |  | 154.3±4.3 | 156.2±4.6^*^ | 155.9±4.5 | 0.020 |
| 14 | 166.4±5.9 | 167.1±5.8 | 166.3±5.2 | 0.064 |  | 155.4±4.3 | 158.4±4.5^**^ | 158.1±4.4^**^ | <0.001 |
| 15 | 169.6±5.3 | 170.5±5.3^*^ | 169.2±4.8 | <0.001 |  | 156.1±4.2 | 159.3±4.3^**^ | 159.1±4.6^**^ | <0.001 |
| 16 | 171.1±5.3 | 172.4±5.3^**^ | 171.1±4.7 | <0.001 |  | 156.6±4.1 | 160.1±4.5^**^ | 159.6±4.6^**^ | <0.001 |
| 17 | 171.8±4.9 | 173.1±5.3^**^ | 172.0±4.8 | 0.003 |  | 157.5±4.1 | 160.6±4.7^**^ | 160.3±4.5^**^ | 0.002 |
| 18 | 172.0±4.6 | 173.1±6.0^*^ | 172.1±5.4 | 0.028 |  | 157.5±3.8 | 160.6±4.4^**^ | 160.4±3.6^**^ | 0.004 |
| Height difference | 47.7±7.2 | 48.9±6.0 | 48.0±7.7 | 0.424 |  | 35.6±5.0 | 38.7±4.7^**^ | 38.5±6.3^**^ | <0.001 |

Note:  ^*^ *P*<0.05，^**^ *P*<0.01, Height difference: height at 18 – the height at 7

**Appendix Section: The fitting process of Preece-Baines model 1**

The Preece-Baines 1 (PB1) function contains five mathematical parameters:

$$h=h_{1}-\frac{2\left( h_{1}-h_{\theta} \right)}{(\exp\left[ s_{0}\left( t-\theta\right) \right]+\exp\left[ s_{1}\left( \left( t-\theta\right) \right] \right))} (1)$$

where h is the height (cm) at time t (years old), h_1_ is the final height (the height at 18 years old), h_θ_ is the height at t =θ, θ is the time constant, and s_1_ and s_0_ are the rate constants. The value of h_θ_ andθwere determined based on previous studies[1-3] (boys: θ=13.5 years old，hθ=145cm; girls:θ=11.5 years old，hθ=130.3cm). The initial value of s_1_ and s_0_ was set as 1 and 0.1, and the maximum likelihood ratio was used to optimize the value. The process of optimization was performed 1000 times. The PB1 function was fitted to height for each annual survey using nonlinear regression.

The acceleration function which can be obtained by differentiating dh/dt, is

$$\frac{d^{2}h}{dt^{2}}=\frac{ds}{dt}\left( h_{1}-h \right)+s\left( -\frac{dh}{dt} \right) (2)$$

The dh/dt is the derivative of height with respect to time, t, i.e velocity. The velocity is

$$\frac{dh}{dt}=s\left( t \right)\cdot\left( h_{1}- h \right) (3)$$

The ds/dt is the derivative of s with respect to time, t, and can be calculated based on

$$\frac{ds}{dt}=\gamma\cdot\left( s_{0}-s \right)\left( s-h_{0} \right) (4)$$

The acceleration function is equal to

$$\frac{d^{2}h}{dt^{2}}=\left( h_{1}-h \right)\left\{ \gamma\left[ \left( s_{0}+s_{1} \right)s-s_{0}s_{1}-s^{2} \right]-s^{2} \right\} (5)$$

The acceleration function simplifies in the special case of PB1 model (when γ=1) to the expression

$$\frac{d^{2}h}{dt^{2}}=\left( h_{1}-h \right)\left[ -2s^{2}+\left( s_{0}+s_{1} \right)s-s_{0}s_{1} \right] (6)$$

The age at peak height velocity or take-off was calculated when the acceleration function is set equal to zero. The equation simplifies by ignoring the case of h = h _1_ to the expression

$${-2s}^{2}+\left( s_{0}+s_{1} \right)s-s_{0}s_{1}=0 (7)$$

For solving s, the equation simplifies to the expression

$$s=\frac{1}{4}\left( s_{0}+s_{1} \right)\pm\sqrt{\left[ \frac{1}{4}\left( s_{0}+s_{1} \right) \right]^{2}-\frac{1}{2}s_{0}s_{1}} (8)$$

Equation 5 can be further manipulated into the form

$$\frac{d^{2}h}{dt^{2}}=\left( h_{1}-h \right)\left[ s^{2}\left( -1-\gamma\right)+s\gamma\left( s_{0}+s_{1} \right)-\gamma s_{0}s_{1} \right] (9)$$

The general solution to a quadratic equation is applied after setting the right-hand side

to zero and ignoring the trivial solution when h = h_1_.

$$s=\frac{-\gamma\left( s_{0}+s_{1} \right)\pm\sqrt{\gamma^{2}\left( \left( s_{0}-s_{1} \right)^{2}+4\gamma s_{0}s_{1} \right)}}{-2-2\gamma} (10)$$

Substituting s from Equation (10) into Equation (11),

$$s=\frac{s_{0}exp(s_{0}(t-\theta))+s_{1}exp(s_{1}(t-\theta))}{exp(s_{0}(t-\theta))+exp(s_{1}(t-\theta))} (11)$$

and solving for t,

$$t=\theta+\frac{{log}_{e}(-\frac{(s-s_{1})}{(s-s_{0})})}{s_{0}-s_{1}} (12)$$

The calculation of time (age) at PHV can be calculated.

When the time of PHV and take-off is known, substituting t into Equation (1) or (2), thus height at PHV and take-off can be calculated. When the height at PHV and take-off is known and s is known, the velocity at PHV and take-off can be calculated. The duration of the height spurt was calculated by age at PHV minus age at take-off.

The standard deviation was used to choose the best model. The standard random error is required to be less than the expected measurement error of height. The growth curve of each child was plotted to determine whether the fit of the PB1 model was acceptable.

Reference.

1. Soliman, A., et al., Advances in pubertal growth and factors influencing it: Can we increase pubertal growth? Indian J Endocrinol Metab, 2014. 18(Suppl 1): p. S53-62.

2. Cao, Z., L.L. Hui, and M.Y. Wong, New approaches to obtaining individual peak height velocity and age at peak height velocity from the SITAR model. Comput Methods Programs Biomed, 2018. 163: p. 79-85.

3. Boeyer, M.E., et al., Estimating peak height velocity in individuals: a comparison of statistical methods. Ann Hum Biol, 2020. 47(5): p. 434-445.
